# The Long Path of Human Placenta, and Its Derivatives, in Regenerative Medicine

**DOI:** 10.3389/fbioe.2015.00162

**Published:** 2015-10-19

**Authors:** Antonietta R. Silini, Anna Cargnoni, Marta Magatti, Stefano Pianta, Ornella Parolini

**Affiliations:** ^1^Centro di Ricerca “E. Menni”, Fondazione Poliambulanza Istituto Ospedaliero, Brescia, Italy

**Keywords:** human term placenta, amniotic and chorionic membranes, umbilical cord, stem cells, clinical trials, immunomodulation, paracrine effect, regenerative medicine

## Abstract

In the 1800s, a baby born with a caul, a remnant of the amniotic sack or fetal membranes, was thought to be lucky, special, or protected. Over time, fetal membranes lost their legendary power and were soon considered nothing more than biological waste after birth. However, placenta tissues have reclaimed their potential and since the early 1900s an increasing body of evidence has shown that these tissues have clinical benefits in a wide range of wound repair and surgical applications. Nowadays, there is a concerted effort to understand the mechanisms underlying the beneficial effects of placental tissues, and, more recently, cells derived thereof. This review will summarize the historical and current clinical applications of human placental tissues, and cells isolated from these tissues, and discuss some mechanisms thought to be responsible for the therapeutic effects observed after tissue and/or cell transplantation.

## A Short History of the Clinical Uses of Human Term Placenta

**Figure 1:**
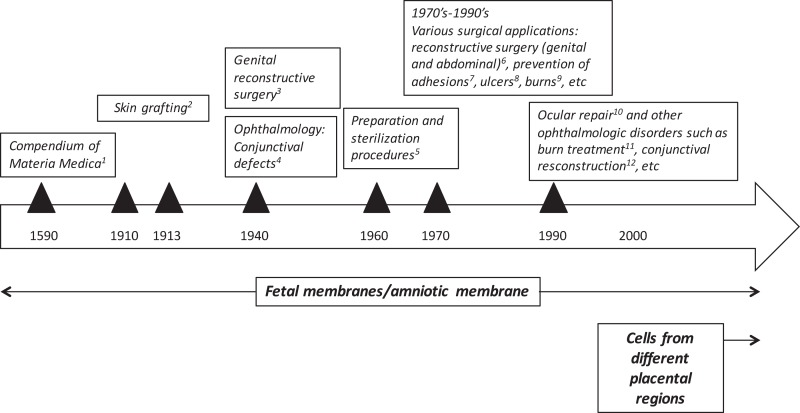
**Historical uses of human term placental tissues and cells derived thereof**. ^1^Chinese medical text published in 1593 by Li Shi-Zhen; ^2^Davis (1910) and Stern (1913) and Sabella (1913); ^3^Brindeau (1934) and Burger (1937); ^4^De Rotth (1940), Sorsby and Symons (1946); Sorsby et al. (1947); ^5^Dino et al. (1965); ^6^Trelford-Sauder et al. (1977); Trelford-Sauder et al. (1978), Silverton et al. (1979); Dhall (1984), Nisolle and Donnez (1992), Georgy and Aziz (1996), Gharib et al. (1996); ^7^Trelford-Sauder et al. (1978); Muralidharan et al. (1991), Young et al. (1991); Arora et al. (1994), Rennekampff et al. (1994); ^8^Troensagaard-Hansen (1950); Bennett et al. (1980), Subrahmanyam (1995); ^9^Gruss and Jirsch (1978) and Bose (1979); ^10^Lee and Tseng (1997); Tseng et al. (1997), ^11, 12^ reviewed in Fetterolf and Snyder (2012).

Human placenta has been traditionally used in Chinese medicine for centuries. The *Compendium of Materia Medica* was published in *1593* by one of the first and greatest biologists and pharmaceutical experts of China, Li Shi-Zhen (Figure 1). This medical text is a Chinese record of substances with medical properties, and it contains a section entirely devoted to the medical uses of human placenta “zi he chi” as a medicine (Young and Benyshek, 2010). At that time, eating the placenta was thought to be beneficial but since then there has been a shift of paradigms in which scientific rationale supports clinical benefit of placental tissues, or derivatives, for treating patients afflicted by a variety of diseases. As discussed below, the earliest reported applications of the placenta (after Li Shi-Zhen’s) were focused on fetal membranes. The first reports showing that the placenta also harbors cells, which could have stem/progenitor properties, ultimately giving rise to their potential use in regenerative medicine, were published many years later (Figure 1), (Bailo et al., 2004; Fukuchi et al., 2004; Igura et al., 2004; In ‘t Anker et al., 2004; Soncini et al., 2007; Troyer and Weiss, 2008).

The first documented use of fetal membranes as a surgical material in skin transplantation came more than 3 decades after the placenta was initially reported to possess medicinal properties. In *1910*, Davis (1910) showed that the use of amniotic membrane (AM) in skin grafting gave superior results when compared to xenograft or cadaveric coverings. Shortly afterwards in *1913*, Stern (1913) and Sabella (1913) reported the use of the AM for treating skin wounds. They applied intact amniotic tissues to skin burns and ulcers and then covered them with dressings. Upon removal of the dressings 2 days later, the authors reported that the amnion had integrated with the patient’s tissues. They also reported lack of infection, a significant decrease in pain, and an increased rate of re-epithelialization of the traumatized skin surface in patients treated with amnion.

More than 20 years passed before another study would report the use of amniotic tissues for wound repair or surgery. Around *1940*, the AM was increasingly being used successfully in different applications. In the late 1930s, Brindeau (1934) and Burger (1937) reported the successful use of amnion for vaginal reconstruction surgery in a patient with Mullerian agenesis. Shortly afterwards, the AM was first applied in ophthalmology to repair conjunctival defects (De Rotth, 1940) and burns (Sorsby and Symons, 1946; Sorsby et al., 1947). Ophthalmology would later go on to be one of the most popular applications of the AM to date.

Following these and other studies, between *1940* and *1970*, a number of clinical trials were published reconfirming the successful use of the AM for skin injuries. In *1940*, the first paper was published describing the use of amnion for the prevention of meningocerebral adhesions following head injury. The authors reported lack of adhesions and lack of rejection 60 days after surgery, and “disappearance” of the amnion after 30 days (Chao et al., 1940). Later that decade, and following Burger’s work on vaginal reconstruction, Kubanyi (1947) used amnion in patients undergoing abdominal surgery with an enterocutaneus fistula secondary to surgery for lysis of adhesions. After closing the small bowel fistula, the small area of the bowel was wrapped with amnion, and notably, patients were discharged on a regular diet only 12 days after surgery. Importantly, Dino et al. (1965) showed that AM from routine deliveries could be sterilized and kept for 6 weeks at 4°C and safely used on acute second degree burns and on skin donor sites (Dino et al., 1965). This was one of the first reports which suggested handling procedures for the AM, which in turn fueled even more interest among clinicians in using the AM for treating skin lesions.

In *1972*, Trelford et al. (1972) used human amnion in sheep, and confirmed previous findings that as a surface graft, amnion was able to reduce pain, decrease infection, and reduce the loss of fluids. Also in 1972, Robson and his colleagues began to study the use of AM for severe burns and skin injuries. Similar to Trelford, they reported notable pain relief and immediate adherence of the AM dressing to the wound. Interestingly, the membranes were easily peeled off 1 week after application and, according to Robson, spontaneous re-epithelialization had occurred underneath the membranes. Later that decade, in *1977*, Trelford-Sauder et al. (1977) reported the successful use of amnion to replace pelvic peritoneum in patients who had exenterative procedures. Following these and other observations, there was an exponential increase of published clinical studies, which reconfirmed the successful application of fetal membranes in diverse clinical indications, including burns (Gruss and Jirsch, 1978; Bose, 1979), ulcers (Troensagaard-Hansen, 1950; Bennett et al., 1980; Subrahmanyam, 1995), surgical reconstruction of the vagina (Dhall, 1984; Nisolle and Donnez, 1992; Georgy and Aziz, 1996), abdominal surgery (Trelford-Sauder et al., 1978; Silverton et al., 1979; Gharib et al., 1996), and other surgical procedures for the prevention of adhesions (Trelford-Sauder et al., 1978; Muralidharan et al., 1991; Young et al., 1991; Arora et al., 1994; Rennekampff et al., 1994).

The *1990s* can be considered the beginning of modern history on the use of AM in ophthalmology (Dua et al., 2004). In this decade, Dr. Tseng, an ophthalmologist from Miami, applied for Human Cell Tissue Products (HCT/P) regulatory status for the use of amniotic tissues in ocular repair. In 1999, Dr. Tseng’s proposal was rejected by the US Food and Drug Administration’s (FDA) tissue reference group stating “Amniotic membrane for ocular surface reconstruction is considered a tissue under the current code of federal regulations (CFR) at 21 CFR Part 1270, but the intended use would be non-homologous when 21 CFR Part 1271 becomes effective” (Lee and Tseng, 1997; Tseng et al., 1997). Dr. Tseng appealed the ruling and 1 year later, the FDA reversed the prior ruling stating that “Allogeneic amniotic membrane for ocular repair is considered to be a section 361 HCT/P if the product’s advertising is restricted to homologous use for wound covering.” The two main points made by the FDA were that it had to be used as a covering and must be acellular, whereas “Amniotic membrane cultured with stem cells for ocular repair is considered to be a biologic product subject to Investigational New Drug and Biologic License Application.” From that point on, the use of AM in ophthalmic surgery catapulted. Nowadays, there are over 45,000 applications used by the ophthalmologists, including conjunctival reconstruction, burn treatment, glaucoma surgery, and wound healing (Fetterolf and Snyder, 2012).

## Current Clinical Applications of Placenta

The twenty-first century marks another turning point in which the use of cells isolated from different placental regions are being progressively more investigated and used for their therapeutic potential. These studies have paved the way for what are now considered established clinical uses and investigative clinical trials. Placental tissues, and cells derived thereof, are nowadays interesting therapeutic biomaterials currently used in the clinic. The renewed interest in using the AM in a number of applications has been favored by the development of improved techniques for both membrane cryopreservation (Lee and Tseng, 1997) and advanced isolation and culture procedures for cells derived thereof.

### Established Clinical Applications

Recent advances in tissue preservation techniques, accompanied by evidence of therapeutic effects, have resulted in commercially available AM products for use in patients. A repertoire of data shows that these products promote rapid and complete healing of wounds.

Without a doubt, the AM is widely utilized in the field of ophthalmology. The AM is routinely used as a graft, spread onto the ocular surface to treat epithelial defects or ulcers, or as a bandage to cover the ocular surface to promote healing. An infinite number of ocular pathologies are being treated with the AM, such as corneal epithelial defects, corneal ulcers, glaucoma, pterygium, and bullous keratopathy (Dua et al., 2004; Liu et al., 2010; Riau et al., 2010; Kesting et al., 2014). Furthermore, the AM is also making its way in dermatology, where it can be used to cover burns and to treat chronic ulcers (Lo and Pope, 2009), and in multiple surgical procedures, some of which already mentioned, and others, such as the prevention of post-operative adhesions. The increasing number of case studies will surely support the establishment of a routine use also in the latter two applications.

### Clinical Trials

**Figure 2:**
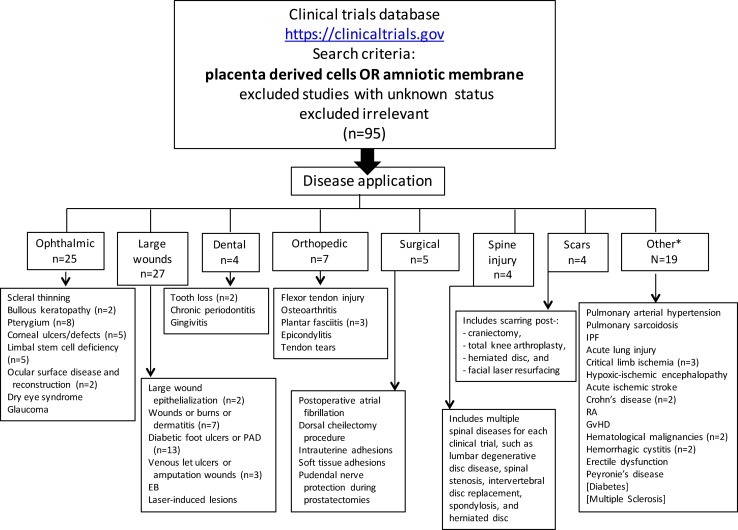
**Registered clinical trials using placenta-derived cells or amniotic membrane**. The search was performed in June 2015 on the U.S. National Institutes of Health clinical trials website using: placental-derived cells OR amniotic membrane, which resulted in 240 clinical trials. After excluding those with unknown status (which resulted in 196) and irrelevant ones, the search resulted in 95 clinical trials. For each disease application specified in small boxes, *n* = 1 unless otherwise specified. PAD, peripheral arterial disease; EB, epidermolysis bullosa; IPF, idiopathic pulmonary fibrosis; RA, rheumatoid arthritis; GvHD, graft versus host disease *Includes two published clinical trials not registered on NIH website (diabetes and multiple sclerosis).

At the time this review was written, 95 clinical trials evaluating placental cells or AM (Figure 2; Tables 1–5) were registered on the NIH Clinical Trials website (https://clinicaltrials.gov).

**Table 1:** **Clinical trials using placenta-derived cells, fetal membranes, or derivatives in ophthalmic disorders**.

Condition	Clinical trial ID	Phase	Materials used	Status	Sponsor	Results/status or remarks
Scleral thinning	NCT00801073	II/III	AM graft	Enrolling	Federal University of São Paulo, Brazil	Primary Completion Date: February 2005, last verified December 2008 (de Farias et al., 2014)
Bullous keratopathy	NCT01926535	II	AM graft	Completed	Universidad de Valparaiso, Chile	None available, Study Completion Date: June 2012
	NCT00659308	ns	AM	Completed	Federal University of São Paulo, Brazil	Study Completion Date: June 2007, last verified January 2008 (Paris Fdos et al., 2013)
Pterygium or ocular surface cicatrizing diseases or keratitis	NCT02102776	ns	AM	Not yet recruiting	Shiyou Zhou, Sun Yat-sen University, China	Estimated Study Completion Date: September 2016
	NCT01319721	ns	AM graft	Completed	Shiyou Zhou, Sun Yat-sen University, China	Results available online (https://clinicaltrials.gov), Study Completion Date: June 2014
	NCT00457223	II/III	AM	Completed	Chulalongkorn University, Thailand	None available, study completed in 2007
	NCT00802620	I	AM	Enrolling	Federal University of São Paulo, Brazil	None available, Primary Completion Date: June 2005, last verified December 2008
	NCT02015000	ns	AM	Recruiting	National Taiwan University Hospital, Taiwan	Estimated Study Completion Date: November 2014, last verified December 2013
	NCT00383825	ns	AM	Completed	Baskent University, Ankara, Turkey	None available, Estimated Study Completion Date: December 2004, last verified October 2006
	NCT00344201	I	AM	Completed	Singapore National Eye Centre, Singapore	None available, Study Completion Date: January 2008
	NCT02116062	ns	AM	Recruiting	University Hospital, Strasbourg, France	Estimated Study Completion Date: March 2016
Corneal wounds or ulcers or epithelial defects	NCT00915759	ns	ProKera^®^	Active	Walter Reed National Military Medical Center, Washington, DC, USA	Estimated Study Completion Date: December 2014, last verified July 2014
	NCT02168790	0	Amnioclip ring system	Completed	Klinikum Chemnitz gGmbH, Germany	None available, Study Completion Date: August 2013
	NCT00238862	ns	AM	Completed	King Khaled Eye Specialist Hospital, Riyadh, Saudi Arabia	None available, study completed in 2007
	NCT02395952	ns	ProKera^®^, Ambiodisk	Not yet recruiting	Milton S. Hershey Medical Center, PA, USA	Estimated Primary Completion Date: February 2018
	NCT02148016	I/II	AM	Recruiting	Sun Yat-sen University, China	Estimated Study Completion Date: September 2014, last verified May 2014
Limbal stem cell deficiency	NCT01377311	I	AM	Terminated	National Taiwan University Hospital, Taiwan	Study terminated since technique not used in patients, last verified June 2011
	NCT00736307	I/II	AM	Completed	Royan Institute, Tehran, Iran	None available, Study Completion Date: October 2009, last verified April 2010
	NCT01562002	I/II	AM	Completed	Instituto Universitario de Oftalmobiología Aplicada, Spain	None available, Study Completion Date: December 2014, last verified January 2015
	NCT01619189	II	AM	Ongoing	Centre Hospitalier National d’Ophtalmologie des Quinze-Vingts, France	Primary Completion Date: December 2014, last verified February 2015
	NCT00491959	I	AM	Terminated	National Taiwan University Hospital, Taiwan	Study terminated due to unstable cell sheet quality, thus this technique was not used on patients
Ocular surface disease and reconstruction	NCT00348114	II	AM	Completed	Singapore National Eye Centre, Singapore	This study has suspended participant recruitment since 2006
	NCT01341223	ns	AM	Recruiting	National Taiwan University Hospital, Taiwan	Estimated Primary Completion Date: March 2016, last verified March 2012
Dry eye syndrome	NCT02369861	I	ACCS	Recruiting	Stemnion, Inc., Pittsburgh, PA, USA	Estimated Study Completion Date: December 2015
Glaucoma	NCT01551550	II/III	AM graft	Recruiting	Tissue Tech™ Inc., Miami, FL, USA	Estimated Study Completion Date: August 2015

*The search status as of June 2015 registered on the ClinicalTrials.gov site of U.S. National Institutes of Health (https://clinicaltrials.gov)*.

*ns, not specified; AM, amniotic membrane; ACCS, amnion-derived Cellular Cytokine Solution (Stemnion, Inc.); ProKera^®^ (Tissue Tech™ Inc.) is a device made by a piece of amniotic membrane tissue in between two rings made out of flexible material*.

**Table 2:** **Clinical trials using placenta-derived cells, fetal membranes, or derivatives in large wounds**.

Condition	Clinical trial ID	Phase	Materials used	Status	Sponsor	Results/status or remarks
Large wound epithelialization	NCT01824381	I	AM	Recruiting	Red de Terapia Celular, Spain	Estimated Study Completion Date: July 2015, last verified April 2013
	NCT01948934	I	AM	Recruiting	Fundacion para la Formacion e Investigacion Sanitarias de la Region de Murcia, Spain	Estimated Study Completion Date: June 2015, last verified March 2014
Wounds or burns or dermatitis	NCT00674999	II/III	Amnion	Withdrawn	The University of Texas Medical Branch, Galveston, TX, USA	Study was withdrawn prior to enrolling due to skin bank being destroyed by hurricane
	NCT00592189	0	Amnion	Completed	The University of Texas Medical Branch, Galveston, TX, USA	None available, Study Completion Date: June 2014, last verified June 2014
	NCT00886470	I/II	ACCS	Terminated	Stemnion, Inc., Pittsburgh, PA, USA	This study was terminated early due to slow accrual of patients
	NCT01715012	II	ACCS	Terminated	Stemnion, Inc., Pittsburgh, PA, USA	This study was terminated early due to enrollment futility
	NCT02389777	II	ACCS	Not yet recruiting	Stemnion, Inc., Pittsburgh, PA, USA	Estimated Study Completion Date: March 2016
	NCT01714973	I	ACCS	Active	Stemnion, Inc., Pittsburgh, PA, USA	Estimated Study Completion Date: October 2015
	NCT02314416*	IV	Amniotic stem cells	Terminated	Georgia Regents University, Augusta, GA, USA	This study has been withdrawn prior to enrollment
Diabetic foot ulcers or peripheral arterial disease or diabetes	NCT02344329	IV	Amnion	Recruiting	University of North Dakota, USA	Estimated Study Completion Date: August 2016, last verified January 2015
	NCT01859117	I	PDA002	Active	Celgene Corporation, NJ, USA	Ongoing, not recruiting, estimated completion data June 2016
	NCT02460081	II	PDA002	Recruiting	Celgene Corporation, NJ, USA	Estimated Study Completion Date: September 2017
	NCT02264288	II	PDA002	Recruiting	Celgene Corporation, NJ, USA	Estimated Study Completion Date: June 2018
	NCT02399826	ns	AM	Recruiting	Lower Extremity Institute for Research and Therapy, OH, USA	Estimated Study Completion Date: January 2016
	NCT02461641	ns	NuShield, Affinity	Recruiting	NuCel, LLC, Birmingham, AL, USA	Estimated Study Completion Date: September 2016
	NCT02209051	IV	AMNIOEXCEL	Ongoing	Derma Sciences, Inc., NJ, USA	Estimated Study Completion Date: August 2015
	NCT01693133	ns	EpiFix^®^	Recruiting	MiMedx^®^ Group, Inc., Marietta, GA, USA	Estimated Study Completion Date: July 2015, last verified March 2015
	NCT01552499	ns	EpiFix^®^	Completed	MiMedx^®^ Group, Inc., Marietta, GA, USA	Study Completion Date: August 2012, last verified September 2012 (Zelen et al., 2014)
	NCT01657474	ns	EpiFix^®^	Completed	MiMedx^®^ Group, Inc., Marietta, GA, USA	None available, Study Completion Date: November 2013, last verified December 2013
	NCT01921491	ns	EpiFix^®^	Recruiting	MiMedx^®^ Group, Inc., Marietta, GA, USA	Estimated Primary Completion Date: May 2015
	NCT02120755	IV	AmnioClear™	Not yet recruiting	Liventa Bioscience, Pennsylvania, USA	Estimated Primary Completion Date: January 2015, last verified April 2014
	NCT02166294	ns	NEOX^®^ CORD 1K	Recruiting	Amniox Medical, Inc., Atlanta, GA, USA	Estimated Study Completion Date: June 2015, last verified July 2014
Venous leg ulcers or amputation wounds	NCT01552447	ns	Epifix^®^	Completed	MiMedx^®^ Group, Inc., Marietta, GA, USA	Study Completion Date: May 2014, last verified November 2013 (Serena et al., 2014)
	NCT02011503	ns	Epifix^®^	Recruiting	MiMedx^®^ Group, Inc., Marietta, GA, USA	Estimated Study Completion Date: January 2016
	NCT00820274	II	AM	Terminated	University Hospital, Limoges, France	Clinical trial encountered difficulties enrolling a sufficient number of patients
Epidermolysis bullosa	NCT02286427	III	AM	Recruiting	Assistance Publique – Hôpitaux de Paris, France	Estimated Study Completion Date: January 2019
Laser-treatment induced lesions	NCT01895374	ns	Bovine AM	Completed	Seoul National University Hospital, South Korea	Study Completion Date: May 2013, last verified July 2013 (Min et al., 2014)

*The search status as of June 2015 registered on the ClinicalTrials.gov site of U.S. National Institutes of Health (https://clinicaltrials.gov)*.

*ns, not specified; AM, amniotic membrane; ACCS, amnion-derived Cellular Cytokine Solution (Stemnion, Inc.); NuShield (NuCel, LLC) is a sterilized dehydrated amnion chorion membrane patch, affinity is an aseptically produced, hypothermically stored amniotic membrane patch. AMNIOEXCEL^®^ (Derma Sciences) is a dehydrated human amnion-derived tissue allograft. EpiFix^®^ (MiMedx^®^) is a dehydrated Human Amnion/Chorion Membrane. NEOX^®^ CORD (Amniox Medical) is made from cryopreserved human amniotic membrane and umbilical cord. AmnioClear™ (Liventa Bioscience) is an allograft membrane comprising of the amnion and chorion*.

**Table 3:** **Clinical trials using placenta-derived cells, fetal membranes, or derivatives in dental and orthopedic defects**.

Condition		Clinical trial ID	Phase	Materials used	Status	Sponsor	Results/status or remarks
Dental defects	Tooth loss	NCT01836783	ns	Amnion	Recruiting	University of Alabama at Birmingham, USA	Estimated Study Completion Date: April 2016
		NCT02482987	ns	BioXclude™	Not yet recruiting	Eisenhower Army Medical Center, GA, USA	Estimated Study Completion Date: July 2018
	Chronic periodontitis	NCT02033226	III	AM	Completed	SVS Institute of Dental Sciences, India	None available, Study Completion Date: September 2013, last verified January 2014
	Gingivitis	NCT02071199	I	ACCS	Recruiting	Stemnion, Inc., Pittsburgh, PA, USA	Estimated Study Completion Date: August 2015
Orthopedic defects	Flexor tendon injury	NCT02361814	ns	AM graft	Recruiting	University of Tampere, Finland	Estimated Study Completion Date: December 2016
	Osteoarthritis	NCT02318511	ns	ReNu amniotic allograft	Recruiting	NuCel, LLC, Birmingham, AL, USA	Estimated Study Completion Date: March 2017
	Plantar fasciitis	NCT02427191	II/III	AmnioFix^®^	Recruiting	MiMedx^®^ Group, Inc., Marietta, GA, USA	Estimated Study Completion Date: December 2017
		NCT01659827	ns	AmnioFix^®^	Completed	MiMedx^®^ Group, Inc., Marietta, GA, USA	Study Completion Date: March 2013, last verified December 2013 (Zelen et al., 2013)
		NCT01996111	ns	EpiFix^®^	Terminated	MiMedx^®^ Group, Inc., Marietta, GA, USA	Study terminated due to change in regulatory status
	Epicondylitis	NCT01921569	ns	Micronized dHACM suspension	Terminated	MiMedx^®^ Group, Inc., Marietta, GA, USA	Study terminated by sponsor, used micronized dehydrated human amniotic membrane (dHACM) suspension
	Tendon tears	NCT01708187	ns	Clarix™1k	Terminated	Orthopedic Foot and Ankle Center, OH, USA	Protocol halted due to less than anticipated recruitment

*The search status as of June 2015 registered on the ClinicalTrials.gov site of U.S. National Institutes of Health (https://clinicaltrials.gov)*.

*ns, not specified; AM, amniotic membrane; dHACM, dehydrated human amniotic membrane; PAD, peripheral artery disease; ACCS, amnion-derived Cellular Cytokine Solution (Stemnion, Inc.); BioXclude™ (Snoasis Medical, Denver, CO, USA) is a human amnion chorion allograft. ReNu™ (NuTech Medical) is a bioactive suspension derived from human amnion and amniotic fluid. AmnioFix^®^ (MiMedx^®^) is a composite amniotic tissue membrane. PLX-PAD are PLacental eXpanded adherent stromal cells (produced by Pluristem Inc.), Clarix™1k (Amniox Medical), C-HAM is a cryopreserved Human Amniotic Membrane*.

**Table 4:** **Clinical trials using placenta-derived cells, fetal membranes, or derivatives in surgery, spinal injuries, and scarring**.

Condition		Clinical trial ID	Phase	Materials used	Status	Sponsor	Results/status or remarks
Surgical	Post-operative atrial fibrillation	NCT02193321	I/II	AM patch	Recruiting	University of Arizona, USA	Estimated Study Completion Date: July 2015, last verified July 2014
	Dorsal cheilectomy procedure for Hallux Rigidus	NCT01825356	IV	AM	Recruiting	OrthoCarolina Research Institute, Inc., Charlotte, NC, USA	Estimated Study Completion Date: December 2016, last verified December 2014
	Intrauterine adhesions	NCT02132104	ns	AM graft	Not yet recruiting	Capital Medical University, Beijing, China	Estimated Study Completion Date: November 2016
	Adhesions of soft tissue during the removal of segmental posterior lumbar instrumentation	NCT01357187	ns	AmnioFix^®^	Completed	MiMedx Group, Inc., Marietta, GA, USA	none available, Study Completion Date: May 2014, last verified June 2014
	Pudendal nerve protection during laparoscopic prostatectomies	NCT01832168	ns	AmnioFix^®^	Completed	MiMedx Group, Inc., Marietta, GA, USA	None available, Study Completion Date: June 2014, last verified March 2015
Spinal injuries	Various spinal injuries^a^	NCT02070484	ns	NuCel^®^	Recruiting	OhioHealth, Colombus, OH,USA	Estimated Study Completion Date: February 2016, last verified April 2014
		NCT02381067	ns	NuCel^®^	Recruiting	NuCel, LLC, Birmingham, AL, USA	Estimated Study Completion Date: September 2016
		NCT02023372	ns	NuCel^®^	Recruiting	NuCel, LLC, Birmingham, AL, USA	Estimated Study Completion Date: July 2017
	Spinal stenosis and herniated disk	NCT02380456	ns	EpiFix^®^	Recruiting	Alexander P. Hughes, MD and MiMedx Group, Inc., Marietta, GA, USA	Estimated Study Completion Date: December 2017
Scarring	Scarring post craniectomy	NCT02033824	ns	EpiFix^®^	Recruiting	MiMedx Group, Inc., Marietta, GA, USA	Estimated Study Completion Date: June 2016
	Scarring post total knee arthroplasty	NCT02088567	ns	EpiFix^®^	Completed	MiMedx Group, Inc., Marietta, GA, USA	None available, Study Completion Date: October 2014, last verified March 2015
	Scarring/spinal stenosis/herniated disk	NCT02300909	IV	EpiFix^®^	Recruiting	MiMedx Group, Inc., Marietta, GA, USA	Estimated Study Completion Date: December 2017
	Scarring post laser resurfacing of the face	NCT01995604	ns	EpiFix^®^	Terminated	MiMedx Group, Inc., Marietta, GA, USA	This study was withdrawn prior to enrollment

*The search status as of June 2015 registered on the ClinicalTrials.gov site of U.S. National Institutes of Health (https://clinicaltrials.gov)*.

*ns, not specified; NuCel^®^ (NuCel, LLC) is a bioactive amniotic suspension derived from human amnion and amniotic fluid. EpiFix^®^ (MiMedx^®^) is a dehydrated Human Amnion/Chorion Membrane (dHACM). AmnioFix^®^ (MiMedx^®^) is a composite amniotic tissue membrane*.

*^a^Includes: lumbar degenerative disk disease, spinal stenosis, spondylolisthesis, spondylosis, invertebral disk displacement and degeneration, spinal diseases, bone disease*.

**Table 5:** **Clinical trials using placenta-derived cells, fetal membranes, or derivatives in other disorders**.

Condition		Clinical trial ID	Phase	Materials used	Status	Sponsor	Results/status or remarks
Lung diseases	Pulmonary arterial hypertension	NCT01795950	I	PLX-PAD	Recruiting	Pluristem Inc., Haifa, Israel	Estimated Study Completion Date: September 2016
	Pulmonary sarcoidosis	NCT01440192	I	PDA001	Terminated	Celgene Corporation, NJ, USA	Study terminated by sponsor
	Idiopathic pulmonary fibrosis	NCT01385644	I	Placental MSC	Completed	The Prince Charles Hospital, Brisbane, QLD, Australia	Study Completion Date: May 2013 (Chambers et al., 2014)
	Acute lung injury	NCT02175303	I/II	Decidual stromal cells	Recruiting	Karolinska Institutet, Stockholm, Sweden	Estimated Study Completion Date: December 2017
Ischemic diseases	PAD/peripheral vascular disease/critical limb ischemia	NCT00951210	I	PLX-PAD	Completed	Pluristem Inc., Haifa, Israel	None available, Study Completion Date: October 2011
		NCT01679990	II	PLX-PAD	Recruiting	Pluristem Inc., Haifa, Israel	Estimated Primary Completion Date: December 2015
		NCT00919958	I	PLX-PAD	Completed	Pluristem Inc., Haifa, Israel	None available, Study Completion Date: June 2012
	Severe hypoxic–ischemic encephalopathy in neonates	NCT02434965	II	HPDSC	Not yet recruiting	New York Medical College, NY, USA	Estimated Study Completion Date: December 2019
	Acute ischemic stroke	NCT01310114	II	PDA001	Terminated	Celgene Corporation, NJ, USA	Study terminated by sponsor
Autoimmune diseases	Crohn’s disease	NCT01155362	II	PDA001	Completed	Celgene Corporation, NJ, USA	None available, Study Completion Date: April 2014, last verified July 2014
		NCT01769755	I	PDA001	Completed	Celgene Corporation, NJ, USA	Study Completion Date: November 2014, last verified April 2015 (Mayer et al., 2013)
	Rheumatoid arthritis	NCT01261403	II	PDA001	Terminated	Celgene Corporation, NJ, USA	Enrollment terminated pending additional Phase 1 data
	Graft versus host disease	NCT02172924	I/II	Decidual stromal cells	Not yet recruiting	Karolinska Institutet, Stockholm, Sweden	Estimated Study Completion Date: December 2019
	Multiple sclerosis	Not applicable	–	PDA001	Published study	–	Lublin et al. (2014)
	Diabetes	Not applicable	–	Placenta-derived MSC	Published study	–	Jiang et al. (2011)
Hematological malignancies	Various^a^	NCT00596999	I	HPDSC	Enrolling	Celgene Corporation, NJ, USA	Estimated Study Completion Date: December 2013, last verified November 2007
	Various^b^	NCT01586455	I	HPDSC	Recruiting	New York Medical College, NY, USA	Estimated Study Completion Date: December 2019
Hemorrhagic cystitis	–	NCT02172963	I/II	Decidual stromal cells	Completed	Karolinska Institutet, Stockholm, Sweden	None available, study completed December 2013
	–	NCT02174536	II	Decidual stromal cells	Recruiting	Karolinska Institutet, Stockholm, Sweden	Estimated Study Completion Date: December 2016
Peyronie’s disease	–	NCT02395029	I	PMD-MSC	Completed	Melissa Marchand from Z Urology, Coral Springs, FL, USA	None available, Study completed in March 2015
Erectile dysfunction	–	NCT02398370	I	PMD-MSC	Completed	Melissa Marchand from Z Urology, Coral Springs, FL, USA	None available, Study completed in March 2015

*The search status as of June 2015 registered on the ClinicalTrials.gov site of U.S. National Institutes of Health (https://clinicaltrials.gov)*.

*ns, not specified; AM, amniotic membrane; PMD-MSC, placental matrix-derived mesenchymal stem cells; PLX-PAD are PLacental eXpanded adherent stromal cells (produced by Pluristem Inc.), Clarix™1k (Amniox Medical), HPDSC (human placenta-derived stem cells produced by Celgene Cellular Therapeutics), PDA001 are human placenta-derived adherent cells (Celgene Corporation)*.

*^a^Includes: myelodysplastic syndrome, acute myelogenous/lymphocytic, leukemia, sickle cell disease, beta thalassemia, inborn errors of metabolism, severe combined immunodeficiency disease*.

*^b^Includes: mucopolysaccharidosis I, mucopolysaccharidosis VI, adrenoleukodystrophy, Niemann–Pick disease, metachromatic leukodystrophy, Wolman disease, Krabbe’s disease, Gaucher’s disease, fucosidosis, Batten disease, severe aplastic anemia, Diamond–Blackfan anemia, amegakaryocytic thrombocytopenia, myelodysplastic syndrome, acute myelogenous leukemia, acute lymphocytic leukemia*.

Surely, there has been much progress since the first documented use of fetal membranes in the early 1900s, and there have been significant advances in the preparation of amniotic and chorionic membranes for clinical use. In fact, there are many companies which commercialize fetal membranes and/or their derivatives, such as Bio-Tissue^®^, a subsidiary of TissueTech™ (Prokera^®^, Amniograft^®^, Amnioguard^®^), IOP Ophthalmics (AmbioDisk™, Ambio2™), MiMedx^®^ (Epifix^®^, Amniofix^®^), Liventa Bioscience (AmnioClear™), Amniox Medical (NEOX^®^ CORD 1K, Clarix™1K), and NuCel (ReNu™ amniotic allograft), just to name a few.

Recently published reports have provided consistent evidence of the therapeutic benefit of the AM graft. For example, the AM has been successfully used in patients with bullous keratopathy (NCT01926535, completed, Table 1), and the results suggested that the technique is safe and represents an efficient alternative to the use of therapeutic contact lenses for the relief of ocular pain in these patients (Venegas et al., 2014). In a different study (NCT00659308, completed, Table 1), the AM was shown to be comparable to anterior stromal puncture in the management of pain in patients with bullous keratopathy (Paris Fdos et al., 2013). When tested in patients with scleral thinning, the AM was not as effective as lamellar corneal and scleral transplantation (de Farias et al., 2014), but a phase II/III trial is now enrolling patients for further testing (NCT00801073, Table 1).

The use of a dehydrated human amnion/chorion membrane Epifix^®^ (MiMedx^®^) has been reported to enhance healing and promote complete epithelialization of diabetic foot ulcers (NCT01552499, completed, Table 2) (Zelen et al., 2014) and to reduce wound size and improve healing also in patients with venous leg ulcers, both when compared to standard treatment (NCT01552447, completed, Table 2) (Serena et al., 2014). This same group also investigated the use of Amniofix^®^ (MiMedx^®^), a dehydrated human amniotic/chorionic membrane refined by a micronization process, in patients with plantar fasciitis (NCT01659827, completed, Table 3) (Zelen et al., 2013). They reported reduced pain and improved physical and mental scores (evaluated by a health survey) versus controls. Interestingly, bovine AM (NCT01895374, completed, Table 2) has been tested for the treatment of laser-induced skin wounds, showing once again quicker epithelialization when compared to hydrocolloid-treated wounds (Min et al., 2014), suggesting that xenogenic material can also be used in wound treatment.

As mentioned above, the interest in using cells isolated from different placental regions intensified in the twenty-first century. Different types of placenta-derived cells and factors are being tested in clinical trials. They come from either fetal placental tissues (such as amnion and chorion) or from maternal tissues (such as decidua). For example, *PLX-PAD* (PLacental eXpanded adherent stromal cells produced by Pluristem Inc.) are isolated from human placenta by enzymatic digestion of both maternal and fetal tissues (Ramot et al., 2009; Kranz et al., 2010). PLX-PAD cells have been used in two clinical trials to treat critical limb ischemia (NCT00919958, completed; NCT00951210, completed), and are now being tested for the treatment of intermittent claudication (NCT01679990, recruiting) and pulmonary arterial hypertension (NCT01795950, active) (Table 5). Thus far, no results have been published for the completed trials.

*PDA-001/PDA-002* are culture-expanded mesenchymal-like cells produced by Celgene Cellular Therapeutics. The safety and efficacy of PDA001 cells have been studied in patients with Crohn’s Disease (Mayer et al., 2013) (NCT01155362, completed; NCT01769755, completed), ischemic stroke (NCT01310114, terminated by sponsor), pulmonary sarcoidosis (NCT01440192, terminated by sponsor), and active rheumatoid arthritis (NCT01261403, terminated) (Table 5). Published results obtained from the Phase I trial (NCT01769755) in patients with Crohn’s disease (Mayer et al., 2013) show that out of the six patients treated with low dose and six with high dose, all low dose and two high dose patients responded to the treatment (defined as ≥70 point decrease from baseline in disease activity score). Some minor evidence of infusion-related events, fever, and headache were reported, but no patient withdrew from the study, and PDA001 was well-tolerated (Mayer et al., 2013). A Phase II study (NCT01155362) investigating PDA001 in treatment-resistant Crohn’s disease has just been completed, but results are not yet available. PDA001 cells have also been recently tested in patients with multiple sclerosis (Lublin et al., 2014). The published results showed that in all 16 patients, none developed 5 or more new lesions in 2 consecutive monthly MRI scans during a 6-month follow-up. Some infusion-related adverse events, such as hematoma and swelling, occurred and settled without medication. This study is important in underlining the safety of placental cells in treating patients with multiple sclerosis, and encourages future studies to investigate therapeutic benefit in this physical and mental disabling condition.

PDA002 cells are currently being tested for the treatment of patients with diabetic foot ulcers and peripheral arterial disease (NCT01859117, active; NCT02460081, recruiting; NCT02264288, recruiting) (Table 2). Human placenta-derived stem cells (*HPDSC*, also produced by Celgene Cellular Therapeutics) are obtained from placenta perfusion after removal non-viable and red blood cells and tissue debris. These cells are cryopreserved without culture expansion (Kang et al., 2013). HPDSC will soon be investigated for treating neonates with severe hypoxic–ischemic encephalopathy (NCT02434965, not yet recruiting), and patients with hematological malignancies (NCT00596999, enrolling; NCT01586455, recruiting) (Table 5).

*Placental-derived MSC* are isolated from the placental tissues after the removal of umbilical cord and external membranes by enzymatic digestions (Prince Charles Hospital in Brisbane, Australia) (Brooke et al., 2009). The safety of placental-derived MSC has been studied in patients with idiopathic pulmonary fibrosis (IPF, NCT01385644, completed). The results of this study have recently been published and show that intravenous infusion of 2 million cells per kilogram is safe in patients with moderate to severe IPF. Of note, the possibility of embolization of cells in the compromised pulmonary vascular bed, a major concern during this type of treatment, did not lead to major adverse clinical outcomes. Only minor, transient changes in hemodynamics and gas exchange, and only minor adverse events were observed (Chambers et al., 2014).

*Placenta-derived decidual stromal cells* can be isolated by either trypsin digestion or tissue explants (Karolinska Institutet, Sweden). These cells derive from the decidua parietalis and are of maternal origin (Ringden et al., 2013; Erkers et al., 2015). They have been tested in a pilot study in patients with hemorrhagic cystitis (NCT02172963, completed) and will soon be investigated in a different clinical trial to treat the same disease (NCT02174536, recruiting) (Table 5). A clinical trial is also due to begin for the treatment of patients with Graft versus Host Disease (NCT02172924, not yet recruiting). Moreover, a pilot study is currently recruiting patients with acute lung injury to evaluate the safety of placenta DSCs (NCT02175303, recruiting) (Table 5).

Remarkably, encouraging results have been published for studies using placenta-derived cells in patients with types II diabetes (Table 5) (Jiang et al., 2011). In this Phase I study aimed at evaluating the safety of placenta-derived MSC, 10 patients with type 2 diabetes were given 3 intravenous infusions at the 1-month intervals. The authors reported that the mean insulin requirement was significantly reduced at the 3-month follow-up, and no side effects (fever, chills, liver damage) were documented. This trial could represent a turning point in the use of placental cells, since it is the first to document their use in patients with diabetes. A different study of particular interest was recently published investigating the immunogenicity of placenta decidual stromal cells in combination with AM in an 11-month-old patient with epidermolysis bullosa (Kaipe et al., 2015), showing improved healing of blisters and wounds, but at the same time warranting further investigations on the immunogenicity of these cells.

Notably, other clinical trials have and are currently investigating placental cell derivatives and, in particular *amnion-derived cellular cytokine solution (ACCS)* (Stemnion, Inc., Pittsburgh, PA, USA). ACCS is obtained from amniotic cell culture (Bergmann et al., 2009), and contains factors relevant for wound healing, such as platelet-derived growth factor (PDGF), vascular endothelial growth factor (VEGF), angiogenin, TGF-β2, TIMP-1, and TIMP-2 (Steed et al., 2008). It is currently being tested in patients with radiation-induced dermatitis (NCT01714973, active), and a separate trial is due to begin in patients with UV-induced burns (NCT02389777, not yet recruiting), (Table 2). Moreover, other trials are currently recruiting patients to test ACCS in dry eye syndrome (NCT02369861, recruiting, Table 1), and gingivitis (NCT02071199, recruiting, Table 3). Two trials, the first aimed at investigating ACCS in partial thickness wounds (NCT00886470), and the second in patients with deep burns (NCT01715012), were terminated due to difficulties in enrolling patients (Table 2).

Another placenta-derived product, amniotic membrane extract (AMX), which is based on lyophilized human AM for topical application, is under investigation in persistent corneal epithelial defects and has shown promising results for reducing epithelial defects (Kordić et al., 2013).

## Mechanisms of Action: The Quest for Scientific Rationale

Evidence of long-term survival with no signs of immune reaction was provided many years ago when the AM was used as an allograft under skin (Douglas et al., 1954) or in the peritoneal cavity (Trelford et al., 1974). Subsequently, a glycoprotein from amnion was reported to be responsible for suppressing “foreign body” reactions by acting on lymphocytes and preventing lymphoblastogenesis (McIntyre and Faulk, 1979).

Since then, numerous mechanisms have been put forth, either to explain the therapeutic effects of the intact or decellularized AM or those of isolated placental cells. Nowadays, the clinical potential of placenta-derived cells essentially relies on their paracrine mechanisms able to induce anti-inflammatory responses and re-epithelialization, and also to possess pro- or anti-angiogenic properties. Below we will briefly discuss these aspects.

### Anti-Inflammatory Properties

Among the paracrine actions underlying the anti-inflammatory effect of placenta-derived cells are their interactions with immune cells of innate and adaptive immunity. Indeed, many studies have reported the ability of placenta-derived cells to suppress the proliferation of activated T cells (Bailo et al., 2004; Chang et al., 2006; Wolbank et al., 2007; Prasanna et al., 2010; Kronsteiner et al., 2011a,b), reduce Th1 inflammatory cytokines and induce T regulatory cells (Raicevic et al., 2011; Ohshima et al., 2012; Anam et al., 2013; Parolini et al., 2014; Pianta et al., 2015), and target B lymphocytes (Li et al., 2005; Ma et al., 2012). Furthermore, they can also influence antigen presenting cells by blocking differentiation of monocytes to dendritic cells (Magatti et al., 2009; Tipnis et al., 2010; Kronsteiner et al., 2011a,b; Saeidi et al., 2013; Banas et al., 2014; Abomaray et al., 2015; Donders et al., 2015; Magatti et al., 2015), and induce M2 macrophage differentiation (Manuelpillai et al., 2012; Abumaree et al., 2013; Magatti et al., 2015). Moreover, placenta-derived cells have been shown to inhibit neutrophils (Zhou et al., 2003; Li et al., 2005; Chen et al., 2014), and natural killer cells (Ribeiro et al., 2013; Chatterjee et al., 2014; Li et al., 2015).

Even though the underlying mechanisms are not completely understood, there are numerous studies which have put forth different hypotheses. Herein, we will briefly summarize them, since comprehensive reviews of the interactions between placenta-derived and immune cells have been described elsewhere (Parolini et al., 2009; Parolini et al., 2010; Manuelpillai et al., 2011; Parolini and Caruso, 2011; Prasanna and Jahnavi, 2011; Abumaree et al., 2012; Caruso et al., 2012; La Rocca et al., 2012; Kim et al., 2013; Silini et al., 2013; Insausti et al., 2014). Cells from placental tissues have been shown to produce factors shown to dampen inflammation, such as interleukin (IL)-10 (Kronsteiner et al., 2011a,b; Rossi et al., 2012; Abomaray et al., 2015; Magatti et al., 2015), transforming growth factor (TGF)-β (Liu et al., 2012; Rossi et al., 2012; Pianta et al., 2015), hepatocyte growth factor (HGF) (Najar et al., 2010; Kronsteiner et al., 2011a,b; Raicevic et al., 2011; Yamahara et al., 2014), prostaglandin E2 (PGE2), (Whittle et al., 2000; Chen et al., 2010; Najar et al., 2010; Kronsteiner et al., 2011a,b; Raicevic et al., 2011; Liu et al., 2012; Rossi et al., 2012; Liu et al., 2014; Yamahara et al., 2014; Abomaray et al., 2015), and indoleamine 2,3-dioxygenase (IDO) enzyme (Chang et al., 2006; Rossi et al., 2012; Anam et al., 2013; Donders et al., 2015). Moreover, placenta-derived cells express negative co-signaling proteins B7H3, PD-L1 (CD274), and PD-L2 (CD273), (Petroff and Perchellet, 2010; Tipnis et al., 2010; Kronsteiner et al., 2011a,b; La Rocca et al., 2012; Abumaree et al., 2013; Wu et al., 2014). HLA-G, a molecule known to have immune-regulatory properties through its interactions with immunoglobulin-like transcript (ILT) receptors (ILT-2, ILT-3, ILT-4), (Allan et al., 2000; Hunt et al., 2005), has been reported to be secreted by a variety of placenta-derived cells (Lefebvre et al., 2000; Chang et al., 2006; Banas et al., 2008; Roelen et al., 2009; Kronsteiner et al., 2011a,b; Pratama et al., 2011; Anam et al., 2013; Donders et al., 2015).

On another note, inflammatory cytokines/milieu have been shown to enhance the immunomodulatory properties of placenta-derived cells. For example, interferon (IFN)-γ has been shown to enhance their anti-proliferative properties on PBMC (Chang et al., 2006; Prasanna et al., 2010; Kronsteiner et al., 2011a,b; Donders et al., 2015). IFN-γ has also been shown to increase HLA-G (Lefebvre et al., 2000; Banas et al., 2008; Kronsteiner et al., 2011a,b), PD-L1 and PD-L2 (Banas et al., 2008; Petroff and Perchellet, 2010; Tipnis et al., 2010; Kronsteiner et al., 2011a,b), and PGE2 production by placental cells (Chen et al., 2010). Moreover, IL-1β, a potent inflammatory cytokine, has also been shown to enhance the immune modulatory properties of placental cells, such as through the induction of PGE2 secretion (Mitchell et al., 1993; Fukuda et al., 1999; Pomini et al., 1999; Chen et al., 2010, Phillips et al., 2011), and by enhancing their suppressive activities toward NK cells (Chatterjee et al., 2014).

### Pro- and Anti-Angiogenic Properties

The intact AM has been shown to produce an array of anti-angiogenic factors. This property is epitomized in the use of intact AM for corneal surface reconstruction, where the use of AM decreases vascularization of the ocular surface through the production of anti-angiogenic proteins (Kim and Tseng, 1995; Shao et al., 2004), such as pigment epithelium-derived factor (PEDF), (Kim and Tseng, 1995; Dawson et al., 1999; Shao et al., 2004), tissue inhibitor of metalloproteinase (TIMP)-1 and TIMP-2 (Hao et al., 2000), and thrombospondin-1 (TSP-1), (Zaslavsky et al., 2010). The intact AM has also been reported to have a large amount of ECM proteins (i.e., laminin-1, laminin-5, fibronectin), which are involved in the suppression of neovascularization in the cornea (Fukuda et al., 1999).

Decellularized AM, whereby amniotic epithelial cells are eliminated and the cytokine-rich ECM is retained, has been shown to maintain anti-angiogenic properties (Tseng et al., 2004).

Furthermore, epithelial and mesenchymal cells isolated from the AM have also been shown to produce anti-angiogenic factors, such as TIMP-1, TIMP-2, TSP-1, and endostatin (Rowe et al., 1997; Hao et al., 2000).

On the other hand, pro-angiogenic properties have also been attributed to the AM, which can be considered important contributors to its wound healing and regenerative capabilities. For example, cytokines known to promote angiogenesis have been found in dehydrated human amnion/chorion membrane, such as angiopoietin-2, epidermal growth factor (EGF), basic fibroblast growth factor (bFGF), heparin binding epidermal growth factor (HB-EGF), HGF, platelet-derived growth factor BB (PDGF-BB), placental growth factor (PlGF), and VEGF (Koob et al., 2014b). Recently, pro-angiogenic factors have been found in conditioned medium from MSC isolated from the AM, which was shown to not only limit infarct size but also promote capillary formation at the infarct border zone when injected into infarcted rat hearts (Danieli et al., 2015). These apparently contradictory properties could give rise to the importance of understanding the impact of the microenvironment in determining the pro- or anti-angiogenic abilities of the AM and placental cells.

### Promotion of Epithelialization

Another important, and well-documented, property of the intact AM is its ability to promote re-epithelialization. The intact AM has been used as a basement membrane to promote epithelial cell migration, differentiation, and prevent epithelial cell apoptosis (Dua et al., 2004). In addition, it produces factors that can stimulate epithelialization, such as bFGF, HGF, and TGFβ (Dua and Azuara-Blanco, 1999; Koizumi et al., 2000). The intact AM also produces factors that support the growth and differentiation of stem and progenitor cells (Meller et al., 2000; Meller et al., 2002, Insausti et al., 2010), such as keratinocyte growth factor (KGF) (Casey and MacDonald, 1997), supporting its use as a progenitor cell niche (Tseng et al., 2004).

Furthermore, dehydrated human amnion/chorion membrane, which preserves ECM composition and retains an array of cytokines, chemokines, and growth factors naturally present in the native tissue, was found to preserve re-epithelialization properties (Koob et al., 2014a).

Studies performed on the AM denuded of epithelial cells have shown significantly lower levels of bFGF, HGF, EGF, and KGF, when compared to intact AM, suggesting an epithelial origin of these factors (Koizumi et al., 2000). Among other ECM proteins found in AM, fibronectin, laminins, and collagen IV and VII have also been reported, which can in turn promote epithelial adhesion and migration (Fukuda et al., 1999; Lobert et al., 2010).

Cells of the AM have also been suggested to promote epithelialization through secretion/production of factors, which can direct migration, proliferation, and differentiation of keratinocytes. In particular, human amniotic mesenchymal cells (hAMSC) and epithelial cells (hAEC) can secrete factors crucial for wound healing. hAMSC highly express EGF, a factor known for its role in keratinocyte and fibroblast migration, IL-8 that promotes re-epithelialization by increasing keratinocyte proliferation and migration, and IGF-1 that is involved in wound closure by promoting the growth of endothelial cells, dermal fibroblasts, and keratinocytes (Kim et al., 2012).

High expression of EGF and PDGF has also been reported in hAEC, the latter of which stimulates the chemotaxis and proliferation of fibroblasts, and is a critical regulator of ECM deposition in healing wounds (Jin et al., 2015). Both hAMSC and hAEC have been shown to engraft into the wound area thus potentially enhancing their paracrine effects and, furthermore, they could directly participate in re-epithelialization by their trans-differentiation into keratinocytes (Kim et al., 2012; Jin et al., 2015).

## Concluding Remarks

Although there are still many open questions regarding the *sine qua non* conditions for the clinical use of placental cells (Fierabracci et al., 2015), the increasing number of clinical trials underlines the interest in using them. Ongoing and future studies will be crucial in helping define their molecular mechanisms, and establishing the true value of placental derivatives (AM, cells, or molecules they release). These studies will undoubtedly unveil additional applications in the field of regenerative medicine. Studies on placental derivatives available thus far have surely contributed to the vision of regenerative medicine, not only based on cell replacement but also on the importance of their paracrine effects, which could promote endogenous tissue regeneration.

## Author Contributions

AS, AC, MM, SP, and OP contributed to writing the manuscript, OP gave final approval of the version to be published. All authors read and approved the manuscript.

## Conflict of Interest Statement

The authors declare that the research was conducted in the absence of any commercial or financial relationships that could be construed as a potential conflict of interest.
